# Effect of Low High-Density Lipoprotein Level on Endothelial Activation and Prothrombotic Processes in Coronary Artery Disease—A Pilot Study

**DOI:** 10.3390/ijerph19148637

**Published:** 2022-07-15

**Authors:** Magdalena Lampka, Dorota Olszewska-Słonina, Iga Hołyńska-Iwan, Zofia Grąbczewska, Karolina Obońska, Anna Cwynar, Justyna Stępowska, Karolina Szewczyk-Golec

**Affiliations:** 1 Department of Pathobiochemistry and Clinical Chemistry, Faculty of Pharmacy, Ludwik Rydygier Collegium Medicum in Bydgoszcz, Nicolaus Copernicus University, 87-100 Toruń, Poland; lampka@cm.umk.pl (M.L.); igaholynska@cm.umk.pl (I.H.-I.); anna.cwynar@cm.umk.pl (A.C.); 2 Department of Cardiology and Internal Diseases, Faculty of Medicine, Ludwik Rydygier Collegium Medicum in Bydgoszcz, Nicolaus Copernicus University, 87-100 Toruń, Poland; z.grabczewska@cm.umk.pl (Z.G.); k.obonska@cm.umk.pl (K.O.); 3 Department of Rehabilitation, Faculty of Medicine, Ludwik Rydygier Collegium Medicum in Bydgoszcz, Nicolaus Copernicus University, 87-100 Toruń, Poland; justyna.stepowska@cm.umk.pl; 4 Department of Medical Biology and Biochemistry, Faculty of Medicine, Ludwik Rydygier Collegium Medicum in Bydgoszcz, Nicolaus Copernicus University, 87-100 Toruń, Poland; karosz@cm.umk.pl

**Keywords:** coronary artery disease, high-density lipoprotein, von Willebrand factor, sE-selectin, sICAM-1, sCD40L

## Abstract

High-density lipoproteins (HDL) play an important role in the prevention of atherosclerosis. The aim of the study was to assess the relationship between serum HDL-C concentration and proinflammatory/prothrombic activation in coronary artery disease (CAD) patients. The study group included 27 acute myocardial infarction (AMI) patients and 30 stable angina pectoris (SA) patients. The control group consisted of 23 people without cardiac symptoms. In the AMI and SA groups, a lower HDL-C and a higher LDL-C/HDL-C index were observed. The SA patients had lower total cholesterol, LDL-C, sE-selectin ligand, as well as higher triglycerides and CD40 concentration in comparison with both the control and AMI groups. A higher von Willebrand Factor and intercellular adhesion molecule-1 were found in both study groups. Low HDL-C concentration in the CAD patients may intensify pro-inflammatory endothelial activation and prothrombotic processes. A low concentration of HDL-C and a high value of the LDL-C/HDL-C index seem to be better indices of atherogenic processes than the LDL-C concentration alone.

## 1. Introduction

High-density lipoproteins (HDL) are assumed to perform an important role in the prevention of atherosclerosis. Their atheroprotective effect is complex. The participation of HDL in the reverse cholesterol transport from tissues to the liver is most significant in this process. The non-lipid antiatherogenic HDL effect is associated with its antioxidative, anti-inflammatory and antithrombotic activity [1,2,3,4].

The antioxidative activity of HDL is engaged in the inhibition of low-density lipoprotein (LDL) oxidative modification. The inhibition of LDL oxidation is determined by apoprotein AI (apo AI), the main protein component of HDL and by paraoxonase 1 (aryldialkylphosphatase, EC 3.1.8.1), an enzyme transported in the HDL fraction [4,5]. Oxidized LDL is cytotoxic to the vascular endothelium, causing damage that initiates atherogenesis processes [4]. Measurement of biochemical endothelial damage is possible by evaluating the von Willebrand factor (vWF) released from endothelial cells into the bloodstream [6].

The anti-inflammatory effect of the HDL fraction is manifested in the inhibition of the expression of adhesion molecules, including P-selectin, E-selectin, intercellular adhesion molecule-1 (ICAM-1) and vascular cell adhesion molecule-1 (VCAM-1), on the endothelial surface. It results in the inhibition of adhesion of T-cells and monocytes to the vascular endothelium and in the migration of these cells to the atheromatous focus. Soluble forms of endothelial adhesion molecules (sP-selectin, sE-selectin, sICAM-1, sVCAM-1) released from the surface of activated endotheliocytes into the bloodstream are indicators of the activation of pro-inflammatory processes [4,6,7].

**Figure 1:**
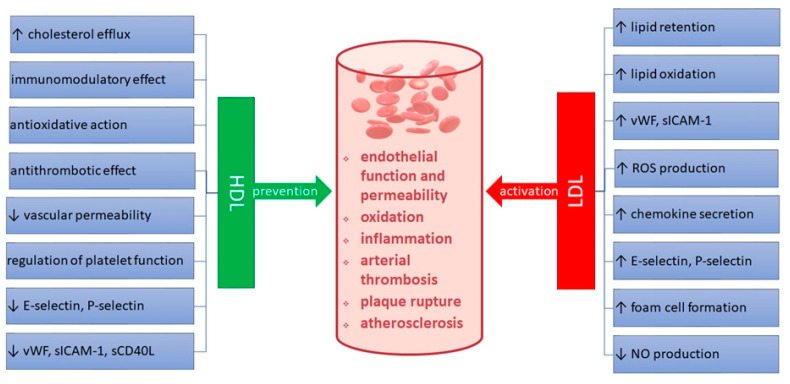
Possible role of high-density (HDL) and low-density (LDL) lipoproteins in the pathogenesis of atherosclerosis and coronary artery disease (CAD). ROS—reactive oxygen species; sCD40L—soluble CD40 ligand; sICAM-1—intracellular adhesion molecule-1; vWF—von Willebrand factor.

HDL also has an antithrombotic effect, which is associated with the inhibition of the blood coagulation process and inhibition of platelet activation [3,4]. It is considered that soluble CD40 ligand (sCD40L) might serve as a new indicator of the severity of prothrombotic process. The sCD40L molecule is a fragment of the transmembrane glycoprotein, CD40L, released from the cell surface into the bloodstream [5]. Both CD40 ligand and its CD40 receptor are coexpressed on cells involved in the atherogenesis process, such as vascular endothelial and vascular smooth muscle cells, activated T lymphocytes, macrophages and platelets. Activation of the CD40/CD40L system initiates an endothelial pro-inflammatory response and exerts prothrombotic effects [8,9]. The possible mechanisms of HDL and LDL action on the atherogenic processes are presented in Figure 1.

The level of HDL in the blood serum can be evaluated based on the level of the HDL cholesterol fraction (HDL-C) or on the concentration of the protein components (apo AI), whereas the LDL level can be assessed by measuring of the LDL cholesterol (LDL-C) fraction [10].

The aim of the study was to evaluate the clinical relation between serum level of HDL-C and the intensity of proinflammatory and prothrombotic endothelial activation, as well as endothelial damage in coronary artery disease (CAD) patients.

## 2. Materials and Methods

### 2.1. Study Group

The study group included 57 CAD patients: 27 acute myocardial infarction (AMI) patients (troponin I concentration: 0.726/6.082 ng/mL, Abott Architect, Poland) and 30 stable angina pectoris (SA) patients treated between September 2013 and December 2014 in the Department of Cardiology and Internal Diseases of Ludwik Rydygier Collegium Medicum in Bydgoszcz, Nicolaus Copernicus University in Torun, Poland. Samples were collected up to 8 h after the onset of symptoms to ensure they serve as a valid estimate of patient lipid profile. The mean age of AMI patients was 61.9 ± 11.2 years and the mean age of patients with SA was 63.3 ± 9.9 years. The diagnosis of CAD was based on prior medical history, clinical examination and contemporary cardiovascular imaging techniques. Before hospitalization, the CAD patients received conventionally recommended drugs, including acetylsalicylic acid (ASA), statins and blood pressure-lowering therapy. In the AMI group, 83% of subjects had ST-elevation myocardial infarction (STEMI) and 17% without ST segment elevation (NSTEMI). The SA group included patients with anginal pain class II according to the Canadian Cardiovascular Society (CCS) classification. The patients were treated with a standard treatment procedure. The control group (C) consisted of 23 volunteers without cardiac symptoms, such as chest pain, nausea, heart palpitations or breathlessness with chest discomfort during an activity. The control subjects were selected from people 45–64 years of age without cardiac symptoms for whom the estimated 10-year risk of fatal cardiovascular disease, assessed with the Systematic Coronary Risk Estimation chart for high-risk regions of Europe [11], did not exceed 4%. The research was conducted in the Department of Pathobiochemistry and Clinical Chemistry of Ludwik Rydygier Collegium Medicum in Bydgoszcz, Nicolaus Copernicus University in Torun, Poland. Each patient gave informed consent for being included in the study. The protocol of retrospective case–control study was approved by the Local Bioethics Committee. All procedures were performed in accordance with the institutional and national ethical standards of the responsible committee on human experimentation and with the Helsinki Declaration of 1975, as revised in 2000 (5).

### 2.2. Laboratory Tests

In the AMI patients, the samples for fasting blood tests were taken from the ante cubital vein during routine checks within 8 h of the cardiac incident. Blood samples from the control group were collected once during routine medical examination at the time of the study. All samples were kept in sterile test tubes (Grainer Bio-one, Kremsmünster Austria).

Concentrations of biochemical markers of endothelial damage were assayed by the ELISA method using a microplate Multiscan Ex reader (Labsystems, Vantaa, Finland). Levels of vWF (Asserachrom vWF, Roche Diagnostics, Warsaw, Poland) were assessed in the citrated plasma, and sCD40L (Human sCD40L, R&D Systems, Warsaw, Poland) in the EDTA plasma. Concentrations of soluble ICAM-1 (human sICAM-1 ELISA, Bender MedSystems, Wien, Austria) and E-selectin (human sE-selectin, Bender MedSystems, Wien, Austria) were assessed in the blood serum. Concentrations of lipids in the blood serum were determined by routine laboratory techniques, namely total cholesterol (TC) and triglycerides (TG) by an enzymatic method, and HDL-C by a direct method (Abbott Architect, Poland). The LDL-C concentration was calculated using the Friedewald formula. The atherogenic indicator value was calculated as a ratio of LDL-C to HDL-C (LDL-C/HDL-C).

### 2.3. Statistical Analysis

Statistical analysis was executed using the STATISTICA 13.1 software (StatSoft, Poland). A power of 80% and alpha level of 0.05 were used to calculate the sample size. The Shapiro–Wilk test was applied for the estimation of the compatibility of the measured parameters’ distribution with normal distribution. The concentration of each determined parameter was presented as median and interquartile range (IQR). The Mann–Whitney and Kruskal–Wallis tests were applied to evaluate the statistical significance of the differences between the study and control groups. A value with *p* < 0.05 was assessed as statistically significant. The relationships between parameters were examined using the Spearman rank correlation coefficients.

## 3. Results

Clinical characteristics and the prevalence of risk factors in the study groups are shown in Table 1. The control and CAD groups were homogeneous regarding risk factors relevant to the development of CAD, including body mass index (BMI), smoking, diabetes, cholesterol and LDL-C levels. The statistically significant difference between the age of the control group and the CAD group results mainly from the difference in age among men from both groups.

Serum lipid profile in both AMI and SA groups was characterized by significantly lower HDL-C concentration and a higher atherogenic index (LDL-C/HDL-C) when compared to the control group. The LDL-C/HDL C index was higher in the AMI group than in the SA group. The SA patients had significantly lower levels of TCH and LDL-C, and significantly higher TG concentrations in comparison with the control and AMI groups (Table 2).

A significant increase in the concentration of endothelial cell markers, including vWF and sICAM-1, was found in both study groups in comparison with the control group. The concentration of vWF in AMI was significantly higher than in SA. The SA patients had a significantly higher concentration of sCD40L in comparison with the control group. No significant difference in the level of sCD40L and sE-selectin was observed between the AMI and control groups. The sE-selectin concentration was lower in the SA group than in the controls and in the AMI group (Table 2).

**Figure 2:**
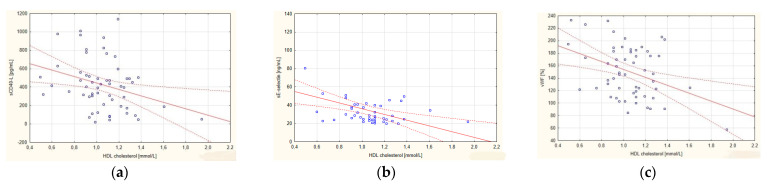
Correlations in the group of coronary artery disease (CAD) patients between the high-density lipoprotein cholesterol (HDL-C) fraction and (**a**) soluble ligand CD40 (sCD40L); (**b**) soluble E-selectin (sE-selectin); and (**c**) von Willebrand factor (vWF). The regression line is marked with a solid line, while the confidence intervals of 0.95 are marked with a dashed line.

**Figure 3:**
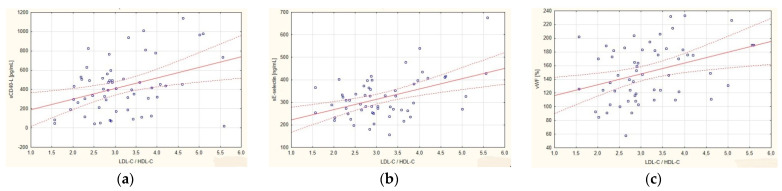
Correlations in the group of patients with coronary artery disease (CAD) between the atherogenic indicator, LDL-C/HDL-C, and (**a**) soluble ligand CD40 (sCD40L); (**b**) soluble E-selectin (sE-selectin); and (**c**) von Willebrand factor (vWF). The regression line is marked with a solid line, while the confidence intervals of 0.95 are marked with a dashed line.

In the group of all patients with CAD, a significant negative correlation was observed between the level of HDL-C and the concentration of vWF, sE-selectin and sCD40L (Figure 2). The value of the atherogenic indicator, LDL-C/HDL-C, positively correlated with the concentration of vWF, sICAM 1 and sCD40L (Figure 3). Concentrations of TCH, LDL-C and TG showed no correlation with any of the determined endothelial cell markers (Table 3).

**Figure 4:**
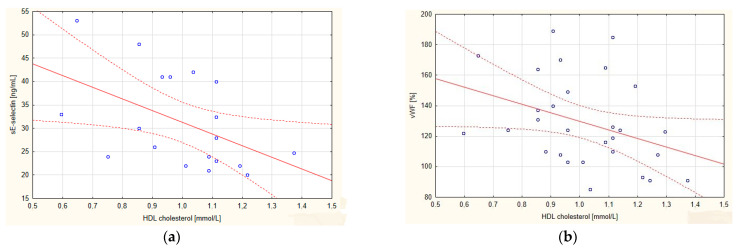
Correlations in the group of patients with stable angina (SA) between the high-density lipoprotein cholesterol (HDL-C) fraction and (**a**) soluble E-selectin (sE-selectin) and (**b**) von Willebrand factor (vWF). The regression line is marked with a solid line, while the confidence intervals of 0.95 are marked with a dashed line.

In the AMI group, HDL-C correlated negatively with vWF, whereas the atherogenic index (LDL-C/HDL-C) and TG correlated positively with sICAM-1. In the SA group, negative correlations of HDL-C with vWF and sE-selectin were evidenced (Table 4, Figure 4). In the control group, the concentration of HDL-C did not correlate with other parameters measured, but the TG concentration correlated negatively with the level of sCD40L (Table 3).

## 4. Discussion

In the present study, a lower concentration of HDL-C and an elevation of endothelial cell markers, including sICAM-1 and vWF, were determined in the patients with AMI and SA, as compared to the control group. An elevated concentration of prothrombotic endothelial marker (sCD40L) was found in both study groups; however, a significant difference versus the controls was shown only in the SA group.

Low concentrations of HDL-C were related to the increased values of the atherogenic index (LDL-C/HDL-C) in both SA and AMI groups in comparison with the control group. Concentrations of TCH and LDL-C in the SA group were significantly lower than in the control group and the AMI group. Statins, taken by most of the examined patients, could have exerted a positive effect on the lipid profile of SA patients. Statin medications reduce LDL-C concentration; however, they do not, or only moderately, affect the HDL-C level [12,13,14].

Low concentrations of HDL-C, observed in all CAD patients, were related to an increase in pro-inflammatory and prothrombotic activity and endothelial damage. This was shown by the negative correlations found between the level of HDL-C and the concentration of endothelial pro-inflammatory activation markers (sE-selectin), endothelial damage markers (vWF) and markers of prothrombotic activity (sCD40L). The influence of low HDL-C levels on the activation of endothelial cells can also be confirmed by the positive correlation between the atherogenic index (LDL-C/HDL-C) value and sICAM-1 concentration.

The composition and conformation of proteins in HDL-C particles are closely related to inflammation, complement activation and immunity [15,16]. Acute inflammation reduces the concentration of plasma HDL-C and changes the protein composition of HDL-C particles [15]. Chronic inflammation, kidney disease and cardiovascular disease are associated with the emergence of new proteins that are not present in HDL-C under physiological conditions, which has been shown to cause HDL-C dysfunction in the anti-inflammatory and antioxidant activities [15,16].

Low HDL levels in male patients with CAD prior to anti-clotting therapy were associated with high sCD40L levels. On the other hand, the normalization of inflammation and a decrease in sCD40L concentration after 45 days of treatment was correlated with increasing HDL-C concentration [17]. Additionally, physiologically structured HDL-C particles inhibit the ICAM-1 expression on inflammation-activated vascular endothelial cells [18].

In the CAD patients, the influence of the atherogenic LDL fraction on the activity and damage of the endothelium was not shown, so the low level of the HDL fraction is supposed to be the main atherogenic factor.

Numerous dysfunctions of HDL have been described in patients with cardiovascular diseases, including reduced capacity of cholesterol efflux from macrophages and other cells, a diminished inhibition of the expression of adhesion molecules on endothelial cells, an impaired stimulation of the bioavailability of endothelial nitric oxide, a limited activity in promoting the survival of endothelial cells, as well as impaired antioxidative effects [19]. Endothelial function and repair are potentiated by HDL [20].

The HDL level in both AMI and SA groups was lower than in the control group, but no significant difference in the value of this parameter between these two pathological conditions was found. Our observations have given rise to the hypothesis that the functionality of the HDL particle might be at least as important as the concentration of HDL-C.

It was found that in patients with AMI, the severity of an ischemic event has an evident impact on HDL function as biomarkers of myocardial infarction extent, such as creatine kinase (CK, EC 2.7.3.2) and its CK-MB isoenzyme, correlate positively with the degree of HDL dysfunction. An acute inflammation, characteristic of AMI, has been shown to reduce the cholesterol efflux properties of HDL. Annema et al. [21] demonstrated that two important atheroprotective functions of HDL, namely cholesterol efflux from macrophage foam cells and the suppression of the expression of inflammation-induced adhesion molecules in endothelial cells, are substantially impaired in STEMI patients.

The soluble cell adhesion molecule expression is intensified by vascular risk factors, including high levels of cholesterol and triacylglycerols and low concentrations of HDL-C. Lupattelli et al. [22] described that low HDL-C levels correlated with higher levels of sVCAM-1 and sICAM-1 in hyperlipidemic subjects free of cardiovascular disease. Calabresi et al. [23] observed increased concentrations of sICAM-1 and sE-selectin in both normolipidemic and hyperlipidemic patients with low HDL-C levels. However, Stanojevic et al. [24] found no relation between low levels of HDL-C and concentrations of sICAM-1, sVCAM-1 and sE-selectin in normolipidemic subjects with CAD.

Elevated vWF concentrations in both clinical groups evidenced endothelial damage, which was more intense in the AMI group. Different states of endothelial disorders are associated with raised plasma levels of vWF. Thus, the vWF measurement has been proposed as a gold standard for the estimation of endothelial damage [6,25,26]. What is important is that vWF participates in the pathological process of the formation of arterial thrombus regarding both the platelet function and coagulation cascade [26,27]. Therefore, it may also be an indicator of increased blood prothrombotic activation.

In patients with CAD, low HDL-C concentration promotes prothrombotic activity. In the present study, this statement was confirmed by the negative correlation between HDL-C and levels of the soluble form of CD40L. The prothrombotic activity of the CD40/CD40L system is expressed by the activation of coagulation process and platelet action, leading to the formation of a blood clot on the surface of atherosclerotic plaque.

The relationship between the level of HDL-C and the activation of the CD40/CD40L system has been also confirmed by other studies. A negative correlation between HDL-C and sCD40L was observed in CAD patients [28] and in subjects with prediabetes [29]. Akinci et al. [30] showed a negative correlation between serum HDL-C and sCD40L levels, as well as a positive correlation between the LDL-C/HDL-C ratio and the sCD40L level in children of parents suffering from metabolic syndrome.

Activation of pro-inflammatory and prothrombotic properties of the CD40/CD40L system may be associated with increased LDL oxidation caused by the reduction of the antioxidant capacity of plasma, induced by decreased HDL-C levels [9]. Oxidized LDL can stimulate the expression of the CD40/CD40L system on endothelial cells. Stimulation of the CD40/CD40L system results in the intensification of adhesion molecule expression on the endothelium and the expression of tissue factor on endothelial and smooth muscle cells. Tissue factor, via the activation of the coagulation system, can also affect the activation of platelets. Activation of pro-inflammatory and prothrombotic processes by the CD40/CD40L system indicates that it is a common link between the early and late processes involved in atherogenesis [9].

In the patients examined in the present study, LDL oxidation processes were not assessed. What is worth mentioning is that no relationship between serum LDL-C levels and the intensity of pro-inflammatory and prothrombotic processes was found. Peng et al. [28] also showed no relationship between concentrations of LDL-C and sCD40L in coronary artery disease. However, sCD40L levels correlated positively with apo B, the major protein component of LDL [28]. Snidermann et al. [31,32] and Vaverkova et al. [33] revealed that apo B is a better risk factor for cardiovascular disease than LDL-C.

Based on the results of the present study, the atherogenic index (LDL-C/HDL-C) seems to be a good indicator of the relationship between the serum lipid profile and pro-inflammatory activation, endothelial damage, as well as prothrombotic processes. Its value depends on the level of both lipoprotein fractions, namely atherogenic LDL and anti-atherogenic HDL. In the present study, the LDL-C/HDL-C index was higher in the AMI group than in the SA group. In all subjects with CAD, this index correlated positively with sICAM-1, vWF and sCD40L.

Interestingly, HDL-C concentration and the atherogenic index (LDL-C/HDL-C) did not affect the concentration of endothelial cell markers in the control group. In the subjects without cardiac symptoms, only a negative correlation between TG and sCD40L concentration was found.

The results of the present study confirmed the pro-atherogenic activity of low HDL levels, assessed as HDL-C concentration. Other authors also indicate that HDL function varies according to the clinical situation [34,35]. Both in the acute phase and in chronic disorders with activation of the inflammatory reaction, an unfavorable transformation of the HDL molecule occurs, leading to the loss of its ability to reverse cholesterol transport and its anti-inflammatory and antioxidant properties. In CAD, a reduction in the ability of HDL to inhibit LDL oxidation was observed. For that reason, the need to introduce new tests to assess the cardiovascular risk has been emphasized in numerous publications. Evaluation of HDL protein composition and/or HDL structure and function has been proposed [2,34,35,36,37].

In the present study, it was shown that low levels of HDL can intensify inflammatory and prothrombotic processes, thus participating in the early and late phases of atherogenesis. It might be assumed that statins taken by the examined patients had a positive effect on qualitative changes in the HDL molecule, although they did not cause an increase in the concentration of this fraction of lipoproteins. However, changes in HDL structure were not examined in the present study.

In addition to the discussed risk factors related to the lipid profile, endothelial dysfunction, which may affect the development of many diseases, such as diabetes, hypertension and CAD, should also be taken into account [38]. The local change in blood flow reduces the oxygen supply and increases the activation and adhesion of platelets. This process results in the augmentation of oxidative stress and inflammatory processes. In people with a genetic burden of endothelial cell dysfunction, it is an additional factor influencing the development of CAD, its severity, course and effectiveness of therapy. The genetic factors include allelic variants of the *NOS3* gene coding for endothelial nitric oxide synthase (eNOS), which lead to endothelial dysfunction and an increased risk of cardiovascular disorders [39]. Therefore, tests assessing endothelial function may be an independent parameter of the risk of cardiovascular disease [38].

The main limitation of the study is the lack of precise information on the types and doses of individual medications taken by the CAD patients. Due to the condition of the patients, it was not possible to obtain accurate data in this regard. The variety of medications used, including ASA, statins and blood pressure-lowering therapy, is undoubtedly a confounding factor in our experiment. As was mentioned above, among the drugs used, statins have been established to significantly reduce LDL-C (mean reduction: 41.8 mg/dL), which has been correlated with a decrease in all-cause mortality and major vascular events [40]. Statins have also been found to act via anti-inflammatory, antioxidant and antiplatelet mechanisms [41]. ASA and blood pressure-lowering therapy, including angiotensin II convertase inhibitors, might also modify the levels of biochemical parameters measured in the present study. Thus, due to the inability to analyze the effect of drugs on the parameters of lipid metabolism, the study focused on comparing these parameters between the AMI and SA patients. The small number of patients is another limitation of the study. However, due to the promising results, we decided to present the obtained relationships and correlations.

According to the current guidelines of the European Society of Cardiology, concerning the procedures of dyslipidemia treatment, HDL-C is not a therapeutic target [11]. Nevertheless, the results of the present study might suggest that the development of effective treatment strategies leading to increased levels of the HDL-C fraction may decrease the risk of cardiovascular disease.

## 5. Conclusions

Low concentrations of high-density lipoproteins in CAD patients may promote pro-inflammatory endothelial activation and intensify prothrombotic processes. The CD40/CD40L system may be a contributing factor to these disturbances. According to the results of the present study, low levels of HDL-C and high values of the LDL-C/HDL-C index seem to be better indices of atherogenic processes in CAD patients than LDL-C concentration alone. Additionally, the measurement of vWF, s-ICAM-1 and sCD-40L might be suggested in patients with heart failure to confirm endothelial dysfunction and provide personalized therapy.

## Figures and Tables

**Table 1:** Clinical characteristics of the coronary artery disease (CAD) patients and control subjects without cardiac symptoms (C).

Parameter	CAD (*n* = 57)	C (*n* = 23)	*p*
Gender: women/men	16 (28%)/41 (72%)	13 (57%)/10 (43%)	0.115/0.240
Age (years)	62.6 ± 10.5 ^	54.3 ± 13.2 ^	0.003
Age: women/men (years)	61.1 ± 13.8 ^/63.3 ± 9.1 ^	57.3 ± 9.0 ^/45.8 ± 12.9 ^	0.235/0.001
BMI (kg/m^2^)	26.69 ± 4.44 ^	25.08 ± 3.01 ^	0.189
BMI: women/men (kg/m^2^)	25.27 ± 4.17 ^/27.01 ± 2.87 ^	24.55 ± 3.12 ^/25.81 ± 2.87 ^	0.530/0.531
BMI > 30 kg/m^2^	11 (19%)	0 (0%)	0.039
Hypertension	28 (49%)	2 (9%)	0.014
Diabetes (stable)	7 (12%)	0 (0%)	0.098
Cigarette smoking	23 (40%)	5 (23%)	0.258
Total cholesterol > 5.0 mmol/L	19 (33%)	16 (70%)	0.180
Triglycerides > 2.3 mmol/L	5 (9%)	1 (4%)	0.870
HDL cholesterol < 1.0 mmol/L	26 (46%)	1 (4%)	0.007
LDL cholesterol > 3.0 mmol/L	30 (53%)	20 (87%)	0.185

Footnotes: BMI—body mass index, ^—values are given as mean ± standard deviation, *p*—statistical significance level. The differences between the groups were determined using a non-parametric Mann–Whitney U test (parameters 2–5) or a two-fraction test (parameters 1; 6–13).

**Table 2:** Lipid parameters and endothelial cell markers in the control (C) subjects without cardiac symptoms and in the coronary artery disease (CAD) patients, divided into two subgroups, including acute myocardial infarction (AMI) and stable angina (SA) patients.

	C (*n* = 23) Me/IQR	CAD (*n* = 57) Me/IQR	*p* *	AMI (*n* = 27) Me/IQR	*p* **	SA (*n* = 30) Me/IQR	*p* ***	*p* ****
Total cholesterol (mmol/L)	5.87/1.24	4.70/1.63	**0.008**	5.12/2.53	0.355	4.24/1.39	**0.001**	**0.018**
LDL cholesterol (mmol/L)	3.67/1.12	3.08/1.39	0.119	3.52/1.88	0.763	2.61/0.92	**0.003**	**0.003**
HDL cholesterol (mmol/L)	1.45/0.70	1.06/0.34	**<0.001**	1.06/0.34	**0.001**	1.01/0.22	**<0.001**	0.203
Triglycerides (mmol/L)	1.16/0.62	1.20/0.62	0.953	1.07/0.59	0.224	1.33/0.60	**0.038**	**0.011**
LDL-C/HDL-C	2.46/1.70	2.90/1.22	**0.020**	3.39/1.17	**0.003**	2.80/0.76	**0.002**	**0.003**
vWF (%)	89.0/40.0	147.0/65.5	**<0.001**	182.0/55.0	**<0.001**	124.0/42.0	**<0.001**	**<0.001**
sE-selectin (ng/mL)	30.2/10.0	26.0/17.5	0.111	29.0/15.0	0.953	22.5/32.3	**0.007**	**0.006**
sICAM-1 (ng/mL)	250.0/64.0	311.0/118.5	**0.001**	311.0/140.0	**<0.001**	306.7/121.5	**0.002**	0.366
sCD40L (pg/mL)	139.0/337.0	410.0/322.0	**0.030**	394.0/665.0	0.326	443.5/200.0	**0.047**	0.367

Footnotes: Me—median value, IQR—interquartile range, vWF—von Willebrand factor, sICAM-1—intracellular adhesion molecule-1, sCD40L—soluble ligand CD40, LDL-C/HDL-C—atherogenic indicator, *p*—statistical significance between groups: *—CAD vs. C (Mann–Whitney test); **—AMI vs. C, ***—SA vs. C, ****—AMI vs. SA (Kruskal–Wallis test).

**Table 3:** Correlations between lipid parameters and endothelial cell markers in the coronary artery disease (CAD) patients and control (C) subjects without cardiac symptoms.

	C (*n* = 23)					CAD (*n* = 57)				
	TCH	HDL-C	LDL-C	LDL-C/ HDL-C	TG	TCH	HDL-C	LDL-C	LDL-C/ HDL-C	TG
vWF	r = −0.005 *p* = 0.980	r = −0.080 *p* = 0.717	r = 0.144 *p* = 0.512	r = 0.127 *p* = 0.565	r = −0.046 *p* = 0.833	r = −0.030 *p* = 0.823	**r = −0.263** ***p* = 0.048**	r = 0.070 *p* = 0.604	**r = 0.372** ***p* = 0.004**	r = −0.142 *p* = 0.293
sE-selectin	r = −0.172 *p* = 0.434	r = −0.171 *p* = 0.436	r = −0.048 *p* = 0.830	r = 0.074 *p* = 0.737	r = 0.124 *p* = 0.573	r = −0.288 *p* = 0.053	**r = −0.359** ***p* = 0.014**	r = −0.219 *p* = 0.145	r = 0.104 *p* = 0.491	r = −0.175 *p* = 0.245
sICAM-1	r = 0.118 *p* = 0.591	r = −0.172 *p* = 0.431	r = 0.161 *p* = 0.462	r = 0.217 *p* = 0.320	r = 0.396 *p* = 0.061	r = 0.167 *p* = 0.214	r = 0.004 *p* = 0.977	r = 0.192 *p* = 0.153	**r = 0.314** ***p* = 0.017**	r = 0.166 *p* = 0.219
sCD40L	r = −0.270 *p* = 0.212	r = 0.252 *p* = 0.245	r = −0.205 *p* = 0.349	r = −0.311 *p* = 0.149	**r =** −**0.528** ***p* = 0.010**	r = −0.013 *p* = 0.923	**r = −0.326** ***p* = 0.013**	r = 0.060 *p* = 0.659	**r = 0.271** ***p* = 0.041**	r = 0.054 *p* = 0.659

Footnotes: vWF—von Willebrand Factor, sICAM-1—intracellular adhesion molecule-1, sCD40L—soluble ligand CD40, LDL-C/HDL-C—atherogenic indicator, r—Spearman’s rank correlation coefficient, *p*—level of statistical significance.

**Table 4:** Correlations between lipid parameters and endothelial cell markers in the patients with acute myocardial infarction (AMI) and stable angina (SA).

	AMI (*n* = 27)					SA (*n* = 30)				
	TCH	HDL-C	LDL-C	LDL-C/ HDL-C	TG	TCH	HDL-C	LDL-C	LDL-C/ HDL-C	TG
vWF	**r = −0.410** ***p* = 0.033**	**r = −0.516** ***p* = 0.006**	r = −0.329 *p* = 0.094	r = 0.268 *p* = 0.177	r = 0.181 *p* = 0.366	r = −0.155 *p* = 0.413	**r= −0.394** ***p* = 0.031**	r = −0.114 *p* = 0.547	r = −0.097 *p* = 0.694	r = 0.035 *p* = 0.854
sE-selectin	r = −0.238 *p* = 0.233	r = −0.306 *p* = 0.120	r = −0.214 *p* = 0.284	r = 0.157 *p* = 0.435	r =−0.006 *p* = 0.976	r = −0.436 *p* = 0.062	**r = −0.511** ***p* = 0.025**	r = −0.314 *p* = 0.190	r = 0.104 *p* = 0.491	r = −0.358 *p* = 0.133
sICAM-1	r = 0.212 *p* = 0.288	r = −0.227 *p* = 0.256	r = 0.234 *p* = 0.241	**r = 0.592** ***p* = 0.001**	**r = 0.517** ***p* = 0.006**	r = 0.079 *p* = 0.680	r = 0.171 *p* = 0.365	r = 0.140 *p* = 0.461	r = 0.040 *p* = 0.833	r = −0.045 *p* = 0.814
sCD40L	r = −0.026 *p* = 0.897	r =−0.357 *p* = 0.067	r = 0.033 *p* = 0.870	r = −0.311 *p* = 0.149	r = 0.129 *p* = 0.520	r = 0.069 *p* = 0.716	r = −0.207 *p* = 0.273	r = 0.207 *p* = 0.272	r = 0.259 *p* = 0.167	r = −0.187 *p* = 0.322

Footnotes: vWF—von Willebrand factor, sICAM-1—intercellular adhesion molecule-1, sCD40L—soluble ligand CD40, LDL-C/HDL-C—atherogenic indicator, r—Spearman’s rank correlation coefficient, *p*—level of statistical significance.

## Data Availability

Data can be found on request.

## References

[1] Annema W., von Eckardstein A. (2013). High-density lipoproteins—Multifunctional but vulnerable protections from atherosclerosis. Circ. J..

[2] Huma Bindu G., Rao V.S., Kakkar V.V. (2011). Friends turns foe: Transformation of anti-inflammatory HDL to proinflammatory HDL during acute phase response. Cholesterol.

[3] Nofer J.R., Brodde M., Kehrel B. (2010). High-density lipoproteins, platelets and the pathogenesis of atherosclerosis. Clin. Exp. Pharmacol. Physiol..

[4] Kosmas C.E., Martinez I., Sourlas A., Bouza K.V., Campos F.N., Torres V., Torres V., Montan P.D., Guzman E. (2018). High-density lipoprotein (HDL) functionality and its relevance to atherosclerotic cardiovascular disease. Drugs Context.

[5] Yin K., Chen W.J., Zhou Z.G., Zhao G.J., Lv Y.C., Ouyang X.P., Yu X.H., Fu Y., Jiang Z.S., Tang C.K. (2012). Apolipoprotein A-1 inhibits CD40 proinflammatory signaling via ATP-binding cassette transporter A1-mediated modulation of lipid raft in macrophages. J. Atheroscler. Thromb..

[6] Constans J., Conri C. (2006). Circulating markers of endothelial function in cardiovascular disease. Clin. Chim. Acta.

[7] Tretjakovs P., Jurka A., Bormane I., Mikelsone I., Elksne K., Krievina G., Reihmane D., Verbovenko J., Bahs G. (2012). Circulating adhesion molecules, matrix metalloproteinase-9, plasminogen activator inhibitor-1, and myeloperoxidase in coronary artery disease patients with stable and unstable angina. Clin. Chim. Acta.

[8] Moreira M.C.S., Pinto I.S.J., Mourão A.A., Fajemiroye J.O., Colombari E., Reis Â.A.S., Freiria-Oliveira A.H., Ferreira-Neto M.L., Pedrino G.R. (2015). Does the sympathetic nervous system contribute to the pathophysiology of metabolic syndrome?. Front. Physiol..

[9] Tasci I., Dogru T., Sonmez A., Genc H., Kilic S., Olgun A., Gok M., Erdem G., Erikci S. (2006). Soluble CD40 ligand levels in otherwise healthy subject with impaired fasting glucose. Mediat. Inflamm..

[10] Reiner Z., Catapano A.L., De Backer G., Graham I., Taskinen M.R., Wiklund O., Agewall S., Alegria E., Chapman M.J., Durrington P. (2011). ESC Committee for Practice Guidelines (CPG) 2008–2010 and 2010–2012 Committees, ESC/EAS guidelines for the management for dyslipidaemias, The task force for the management of dyslipidaemias of the European Society of Cardiology (ESC) and European Atherosclerosis Society (EAS), European Association for Cardiovascular Prevention & Rehabilitation. Eur. Heart J..

[11] Mach F., Baigent C., Catapano A.L., Koskinas K.C., Casula M., Badimon L., Chapman M.J., De Backer G., Dalgadok V., Ferencel B.A. (2019). 2019 ESC/EAS guidelines for the management of dyslipidaemias: Lipid modification to reduce cardiovascular risk. Atherosclerosis.

[12] Kei A., Tellis C., Liberopoulos E., Tselepis A., Elisaf M. (2014). Effect of switch to the highest dose of rosuvastatin versus add-on-statin fenofibrate versus add-on-statin nicotinic acid/laropiprant on oxidative stress markers in patients with mixed dyslipidemia. Cardiovasc. Ther..

[13] Hoshiga M., Arishiro K., Nakakaoji T., Myiazaki N., Negoro N., Okabe T., Kohbayashi E., Ishihara T., Hanafusa T. (2010). Switching to aggressive statin improves vascular endothelial functions in patients with stable coronary artery disease. J. Atheroscler. Thromb..

[14] Blanco-Colio L.M., Martin-Ventura J.L., de Teresa E., Farsang C., Gaw A., Gensini G.F., Leiter L.A., Langer A., Martineau P., Egido J. (2008). Atorvastatin decreases elevated soluble CD40L in subjects at high cardiovascular risk. Atorvastatin on inflammatory markers study: A substudy of ACTFAST. Kidney Int..

[15] Ronsein G.E., Vaisar T. (2017). Inflammation, Remodeling and Other Factors Affecting HDL Cholesterol Efflux. Curr. Opin. Lipidol..

[16] Eren E., Ellidag H.Y., Aydin O., Yilmaz N. (2015). HDL functionality and crystal-based sterile inflammation in atherosclerosis. Clin. Chim. Acta.

[17] Obradovic S., Djukanovic N., Todorovic Z., Markovic I., Zamaklar-Trifunovic D., Protic D., Ostojic M. (2015). Men with lower HDL cholesterol levels have significant increment of soluble CD40 ligand and high-sensitivity CRP levels following the cessation of long-term clopidogrel therapy. J. Atheroscler. Thromb..

[18] Tabet F., Vickers K.C., Torres L.F., Wiese C.B., Shoucri B.M., Lambert G., Catherinet C., Prado-Lourenco L., Levin M.G., Thacker S. (2014). HDL-transferred microRNA-223 regulates ICAM-1 expression in endothelial cells. Nat. Commun..

[19] Annema W., von Eckardstein A. (2016). Dysfunctional high-density lipoproteins in coronary heart disease: Implications for diagnostics and therapy. Transl. Res..

[20] Ragbir S., Farmer J.A. (2010). Dysfunctional high-density lipoprotein and atherosclerosis. Curr. Atheroscler. Rep..

[21] Annema W., Willemsen H.M., de Boer J.F., Dikkers A., van der Giet M., Nieuwland W., Muller Kobold A.C., van Pelt L.J., Slart R.H., van der Horst I.C. (2016). HDL function is impaired in acute myocardial infarction independent of plasma HDL cholesterol levels. J. Clin. Lipidol..

[22] Lupattelli G., Marchesi S., Lombardini R., Siepi D., Bagaglia F., Pirro M., Ciuffetti G., Schillaci G., Mannarino E. (2003). Mechanisms of high-density lipoprotein cholesterol effects on the endothelial function in hyperlipidemia. Metabolism.

[23] Calabresi L., Gomaraschi M., Villa B., Omoboni L., Dmitrieff C., Franceschini G. (2002). Elevated soluble cellular adhesion molecules in subjects with low HDL-cholesterol. Arterioscler. Thromb. Vasc. Biol..

[24] Stanojević N.B., Ivanović Z.J., Djurović S., Kalimanovska V.S., Spasić S., Oštrić D.K., Memon L. (2005). Lack of association between low HDL-cholesterol and elevated circulating cellular adhesion molecules in normolipidemic CAD patients and healthy subjects. Int. Heart J..

[25] Lee K., Lip G., Tayebjee M., Foster W., Blann A. (2005). Circulating endothelial cells, von Willebrand factor, interleukin-6, and prognosis in patients with acute coronary syndromes. Blood.

[26] Spiel A., Gilbert J., Jilma B. (2008). Von Willebrand factor in cardiovascular disease, Focus on acute coronary syndrome. Circ. J..

[27] Gragnano F., Sperlongano S., Golia E., Natale F., Bianchi R., Crisci M., Fimiani F., Pariggiano I., Diana V., Carbone A. (2017). The role of von Willebrand Factor in vascular inflammation: From pathogenesis to targeted therapy. Mediat. Inflamm..

[28] Peng D.Q., Zhao S.P., Li Y.F., Li J., Zhou H.N. (2002). Elevated soluble CD40 ligand is related to the endothelial adhesion molecules in patients with acute coronary syndrome. Clin. Chim. Acta.

[29] Genc H., Dogru T., Tapan S., Tasci I., Bozoglu E., Gok M., Aslan F., Celebi G., Erdem G., Avcu F. (2012). Soluble CD40 ligand, soluble P-selectin, and von Willebrand factor level in subjects with prediabetes: The impact of metabolic syndrome. Clin. Chem..

[30] Akinci G., Coskun S., Akinci B., Hekimsoy Z., Bayindir B., Onur E., Ozmen B. (2007). Atherosclerosis risk factors in children of parents with metabolic syndrome. Atherosclerosis.

[31] Sniderman A.D., Williams K., Contois J.H., Monroe H.M., McQueen M.J., de Graaf J., Furberg C.D. (2011). A meta-analysis of low-density lipoproteins cholesterol, non-high density lipoproteins cholesterol, apolipoprotein B as markers of cardiovascular risk. Circ. Cardiovasc. Qual. Outcomes.

[32] Sniderman A.D., Islam S., McQueen M., Pencina M., Furberg C.D., Thanassoulis G., Yusuf S. (2016). Age and Cardiovascular Risk Attributable to Apolipoprotein B, Low-Density Lipoprotein Cholesterol or Non-High-Density Lipoprotein Cholesterol. J. Am. Heart Assoc..

[33] Vaverkova H., Karasek D., Navotny D., Jackuliakova D., Lukes J., Halenka M., Frohlich J. (2009). Apolipoprotein B versus LDL-cholesterol: Association with other risk factors for atherosclerosis. Clin. Biochem..

[34] Eren E., Yilmaz N., Aydin O. (2012). High density lipoprotein and it’s dysfunction. Open Biochem. J..

[35] Choi H.Y., Hafiane A., Schwertani A., Genest J. (2017). High-Density Lipoproteins: Biology, Epidemiology, and Clinical Management. Can. J. Cardiol..

[36] Hafiane A., Genest J. (2013). HDL, Atherosclerosis, and emerging therapies. Cholesterol.

[37] Halcox J.P., Banegas J.R., Roy C., Dallongeville J., De Backer G., Guallar E., Perk J., Hajage D., Henriksson K.M., Borghi C. (2017). Prevalence and treatment of atherogenic dyslipidemia in the primary prevention of cardiovascular disease in Europe: EURIKA, a cross-sectional observational study. BMC Cardiovasc. Disord..

[38] Alexander Y., Osto E., Schmidt-Trucksäss A., Shechter M., Trifunovic D., Duncker D.J., Aboyans V., Bäck M., Badimon L., Cosentino F. (2021). Endothelial function in cardiovascular medicine: A consensus paper of the European Society of Cardiology Working Groups on Atherosclerosis and Vascular Biology, Aorta and Peripheral Vascular Diseases, Coronary Pathophysiology and Microcirculation, and Thrombosis. Cardiovasc. Res..

[39] Severino P., D’Amato A., Prosperi S., Magnocavallo M., Mariani M.V., Netti L., Birtolo L.B., De Orchi P., Chimenti C., Maestrini V. (2021). Potential Role of eNOS Genetic Variants in Ischemic Heart Disease Susceptibility and Clinical Presentation. About J. Cardiovasc. Dev. Dis..

[40] Karmali K.N., Lloyd-Jones D.M., Berendsen M., Goff D.C., Sanghavi D.M., Brown N., Korenovska L., Huffman M.D. (2016). Drugs for primary prevention of atherosclerotic cardiovascular disease: An overview of systematic reviews. JAMA Cardiol..

[41] Hennekens C.H., Schneider W.R. (2008). The need for wider and appropriate utilization of aspirin and statins in the treatment and prevention of cardiovascular disease. Expert Rev. Cardiovasc. Ther..

